# Non-covalent and reversible functionalization of carbon nanotubes

**DOI:** 10.3762/bjnano.5.178

**Published:** 2014-09-30

**Authors:** Antonello Di Crescenzo, Valeria Ettorre, Antonella Fontana

**Affiliations:** 1 Dipartimento di Farmacia, Università “G. d’Annunzio”, Via dei Vestini, 66100 Chieti, Italy

**Keywords:** carbon nanotubes, non-covalent functionalization, π-stacking, reversible dispersion/precipitation

## Abstract

Carbon nanotubes (CNTs) have been proposed and actively explored as multipurpose innovative nanoscaffolds for applications in fields such as material science, drug delivery and diagnostic applications. Their versatile physicochemical features are nonetheless limited by their scarce solubilization in both aqueous and organic solvents. In order to overcome this drawback CNTs can be easily non-covalently functionalized with different dispersants. In the present review we focus on the peculiar hydrophobic character of pristine CNTs that prevent them to easily disperse in organic solvents. We report some interesting examples of CNTs dispersants with the aim to highlight the essential features a molecule should possess in order to act as a good carbon nanotube dispersant both in water and in organic solvents. The review pinpoints also a few examples of dispersant design. The last section is devoted to the exploitation of the major quality of non-covalent functionalization that is its reversibility and the possibility to obtain stimuli-responsive precipitation or dispersion of CNTs.

## Introduction

**Figure 1:**
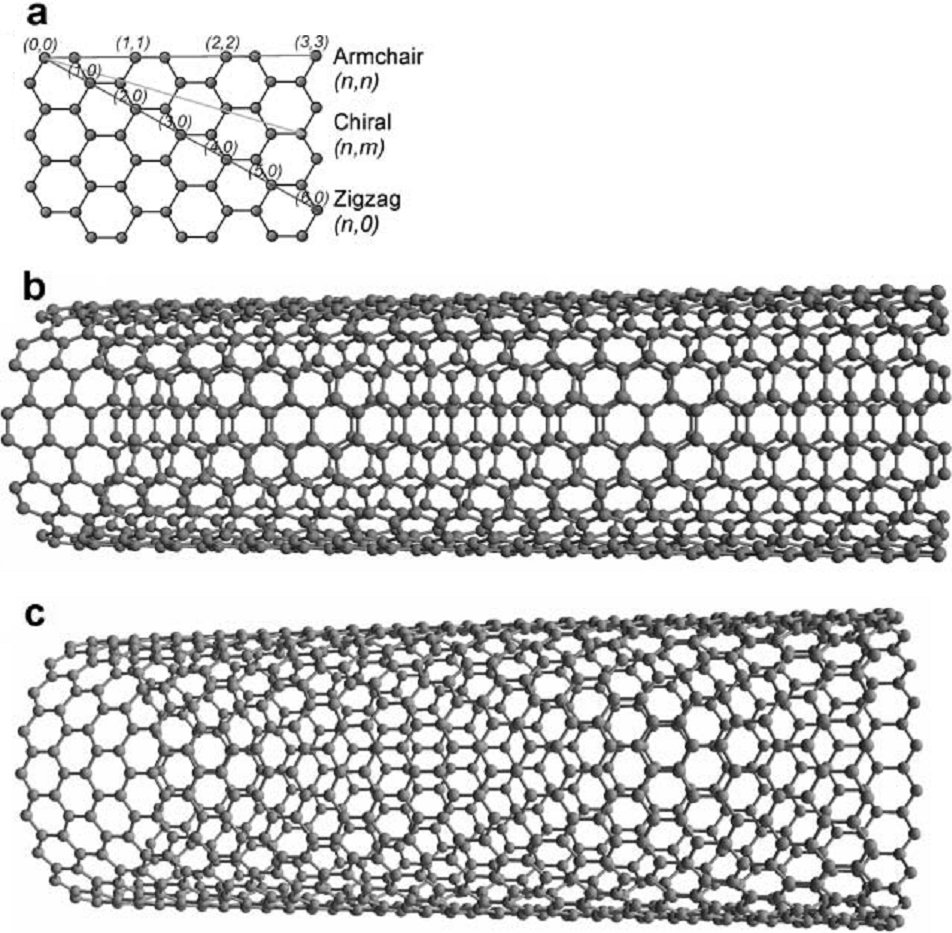
(a) The wrapping vector of a graphene sheet defines the structure (chirality) of a carbon nanotube. Examples of (b) ‘‘armchair’’ and (c)‘‘zig-zag’’ SWCNTs. [1]. – Reproduced by permission of The Royal Society of Chemistry.

Carbon nanotubes (CNTs) are hollow cylindrical tubes with nanometer scale diameters and lengths up to a few micrometers. They are ideally obtained by a rolled up graphene sheet and their diameter, curvature and electronic properties are uniquely defined by the combination of the rolling angle and radius, which is referred to as nanotube chirality (Figure 1) [1]. CNTs made up of a rolled up single graphene sheet and closed at their ends by hemispheric fullerene caps are referred to as single-walled nanotubes (SWCNTs) and their diameter ranges from 0.4 nm to 5 nm [2–3]. Multi-walled carbon nanotubes, made up of several concentric graphene cylinders, are much bigger with diameters from a few to tens of nanometers.

These one-dimensional nanostructures reveal exceptional thermal, electrical, mechanical and optical properties [4], which make them promising candidates for potential applications in various fields. CNTs have found applications in nanotechnology [5], electronics [6], sensors and biosensors [7–9], catalysis [10], energy conversion [11–12] storage devices [13], the delivery field [14–15], and in the polymer field [16] to name a few. However, CNTs applications have been hindered for a long time from their discovery [17] due to their limited solubility and processability. Individual CNTs tend to strongly interact with each other through van der Waals forces reaching ~500 eV per μm of CNT’s length [18] and aggregate into bundles and ropes. In order to counteract these forces and favor CNTs manipulability and solubility mainly two strategies have been adopted: i) covalent functionalization through attachment of molecular pendants to the Csp^2^ backbone [19–20] and ii) non-covalent functionalization by adsorption of molecules onto the nanotube surface [21]. Alternatively, in order to use CNTs for elected applications, encapsulation of molecules in the inner empty cavity of the nanotubes has been exploited [22–24]. To date, several successful strategies have been applied to covalently derivatize CNTs sidewalls. Typical approaches include acid oxidation [25], cycloadditions spanning from the widely known 1,3-dipolar cycloaddition, namely the Prato reaction [26–28] to the Bingel [2 + 1] cyclopropanation [29], radical additions via diazonium salts [30–31] grafting of polymers. Nevertheless the creation of novel covalent bonds disrupt the sp^2^ network of non-derivatized CNTs and may thus alter their inherent properties [30,32]. The prospect of functionalizing CNTs outer surface via non-covalent ways through chemical adsorption of ordered architectures [33–34] and preserve the extended networks of the CNTs seems therefore very attractive.

In this review, starting from several examples of good dispersions and dispersants, we intend to systematically identify the features that allow a molecule to behave as a good CNTs dispersant both in organic and in aqueous solvents via non-covalent functionalization. Once the essential morphological characteristics of a dispersant have been outlined, it will be much easier to conceive novel molecules with additional properties but unchanged functionalities responsible for water or organic solvent solubility or nanotubes debundling. Several recent practical applications for CNTs involved in non-covalent interactions with elected molecules both in aqueous and organic environment are discussed. In particular, a light will be shed on the possibility of creating systems able to respond to elected external chemical or physical stimuli such as pH, temperature or light thanks to the major advantage of non-covalent functionalization, namely its reversibility.

## Review

### Morphological and electronic characteristics of CNTs

Despite we do not want to put forward an exhaustive CNTs description, we think that, in order to evaluate the features that a dispersant should possess to favor CNTs dispersion, it is important to briefly describe CNTs properties. Carbon nanotubes, as well as graphene, are built mainly of Csp^2^ arranged in hexagons but, differently from benzene, carbon atoms in a nanotube are pyramidalized and π-orbitals are misaligned due to the curvature of the tube surface [1] .These conditions confer to nanotubes an intrinsic p-type semiconducting behavior and a reactivity much more pronounced than that of graphite. Accordingly, nanotubes with bigger diameter are less reactive than smaller nanotubes whereas the inner surface of carbon nanotubes appears to be much less reactive than the outer one. These differences manifest themselves as well when non-covalent interactions are concerned. In fact, Tournus et al. [35] showed that wider nanotubes tend to interact easier than narrow nanotubes with benzene due to the better geometric match between a planar molecule such as benzene and the not very pronounced π-orbital misalignment of bigger CNTs.

The chirality of CNTs, that depends on the way carbon hexagons are arranged in the nanotube (see Figure 1), and their diameter affects the conductance, density and honeycomb lattice structure of the tube and allow to divide them in two main types, semiconducting and metallic. The CNTs chirality appears to be severely involved in the dispersion of CNTs. As an example, the chirality of the tubes drives the interactions that nanotubes establish with the surrounding medium with semiconducting tubes favoring donor-acceptor interactions [36–37].

### Non-covalent interactions and hydrophobicity of CNTs

Despite pristine carbon nanotubes possess a π-conjugative structure with a highly hydrophobic surface, they are scarcely soluble in most organic solvents due to the high molecular weight and their tendency to entangle and form 3D networks through persistent van der Waals interactions. Therefore, for CNTs applications, the prime aim is to promote CNTs entanglement by energetic agitation. Generally, agitation is provided by magnetic stirring, reflux, shear mixing, or, most commonly, ultrasonication either mild sonication in a bath or high-power sonication using a tip [16]. Once exfoliated, the simplest stable CNTs dispersions have been achieved by using solvent molecules able to efficiently interact with CNTs such as phenylethyl alcohol [38] or *N*-methylpyrrolidone (NMP) [39]. Indeed NMP has been demonstrated to enter the bundles during sonication and remain strongly bound to the nanotube surface. This leads to an enthalpy of mixing that is approximately zero conferring to the corresponding free energy of mixing a negative value. However, NMP molecules could be removed by heating to 340 °C, leaving perfectly intact nanotubes and demonstrating that NMP was physisorbed via van der Waals interactions onto the nanotube surface. Good and relatively stable dispersions have been obtained also by sonicating CNTs with *N*,*N*-dimethylformamide (DMF) [40], highlighting that a high value of β (the hydrogen bond acceptance basicity), a negligible value of R (the hydrogen bond donation parameter of Taft and Kamlet) and a high value for π* (solvochromic parameter) [41] are necessary, although not sufficient conditions, to ensure a good CNTs dispersion. Landi et al. [42] obtained a dispersion stable for days by sonicating CNTs with *N*,*N*-diethylacetamide (DEA) and imputed the stability of the dispersion to synergistic effective polar and π*-*stacking interactions. As a matter of fact solvents characterized by either dipole–dipole or *π-*orbital overlap interactions may not account for an equivalent dispersion performance, neither equally polar solvents such as acetonitrile (dielectric constant 36.00) and DMSO (dielectric constant 46.71) or 1,2-dichlorobenzene able to give higher π-orbital overlap interactions despite being less polar (dielectric constant 10.36) [43] than DEA.

An alternative strategy to favor CNTs dispersion in organic solvents is to coat CNTs with a dispersant phase, usually a molecule characterized by a high affinity towards nanotube sidewalls and at the same time particularly soluble in the elected solvent. In particular both small molecules and high weight polymers have been used as dispersants.

Aromatic molecules have a strong affinity for graphitic surfaces via π–stacking. The adsorption of different polycyclic aromatic hydrocarbons (PAH) such as pyrene, anthracene, tetracene and phenanthrene on SWCNTs has been extensively investigated. The strong interactions of different anthracene derivatives to shortened SWCNTs has been made clearly evident [44] by changes in both the UV–vis and FTIR spectra and shift in fluorescence spectra. The displacement of attached anthracene by an excess amount of pyrene in THF indicated that the adsorption process is reversible. Instead tetracene and pentacene were investigated by X-ray photoelectron spectroscopy [45]. The more than six times greater adsorption of tetracene with respect to that of phenanthrene was imputed to the nanoscale curvature of the tube surface, and the consequent difference in the contacts between the molecule and the tube surface. The observed shift of the radial breathing mode (RBM) of the Raman band towards high frequency confirmed the strong π–π interactions between PAH and the external SWCNT surface. Wang et al. demonstrated that sorption of phenanthrene and naphtalene to CNTs is correlated to the hydrophobicity of the corresponding PAH [46]. Stable dispersions of SWCNTs in THF have been easily achieved by sonicating CNTs with diazapentacene at room temperature [47] (see Table 1). The adsorption is solely imputable to π-stacking as no solvophobic forces can participate in the interactions with the SWCNT walls. This functionalization leaves virtually intact the inherent properties of SWCNTs, as observed by several photophysical measurements. Porphyrins (see Table 1) demonstrated [48] to physically adsorb onto the CNTs surface and dissolve both individual and bundled SWCNTs in organic solvents. The solid SWCNTs–Zn-protoporphyrin nanocomposite is readily separable from the solution and can be easily redissolved in DMF.

**Table 1:** Some lipophilic molecules used for the dispersion of CNTs in organic solvents.

dispersant (structure or name)	acronym	organic solvent	ref.

anthracene and substituted-anthracene	–	THF	[44]
tetracene and pentacene	–	THF	[45]
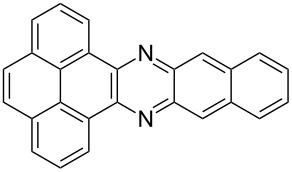	diazapentacene derivative	THF	[47]
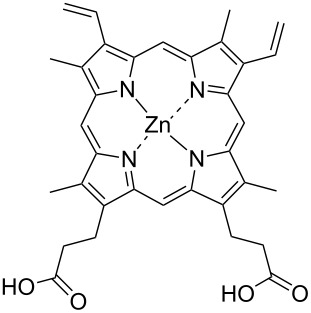	ZnPP	DMF	[48]
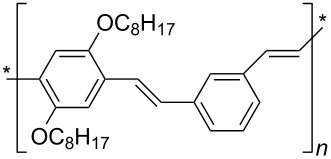	PmPV	toluene	[49]
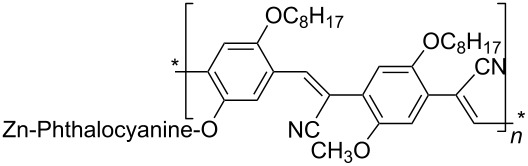	PPV	THF	[50]
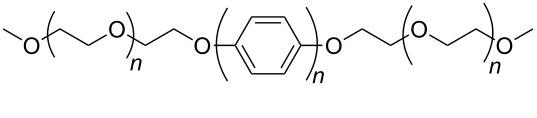	(PEG)_8_(Ph)_6_(PEG)_8_	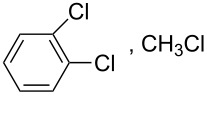	[52]
polystyrene-*b*-polyisoprene		DMF heptane	[53]
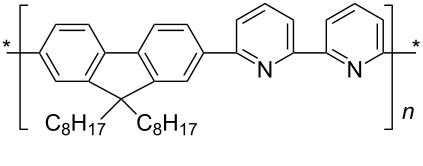	PFO-BPy	toluene, THF	[55]
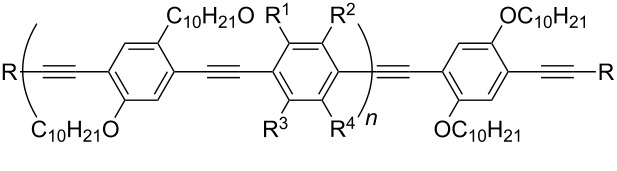	PPE 1	CH_3_Cl	[56]
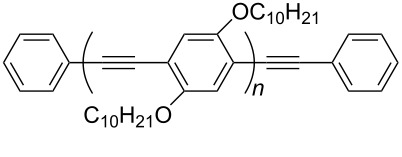	PPE 2	CH_3_Cl	[56]
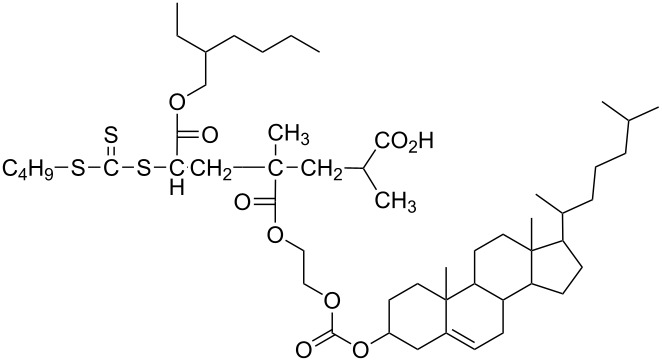	CEM-EHA	THF, toluene, isooctane	[57]
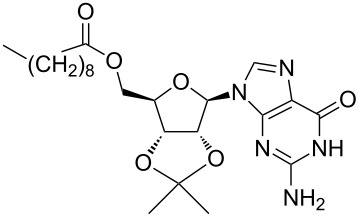	LipoG 1	CHCl_3_	[58]
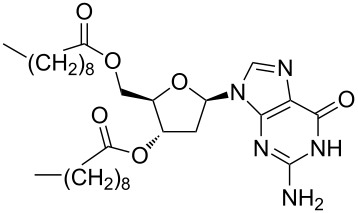	LipoG 2	CHCl_3_	[58]

Typical dispersants are polymers formed by repetitive units of alkyl chains and aromatic moieties which exploit both π-stacking to the CNT surface and van der Waals interactions between the hydrophobic nanotube surface and alkyl tails. It has been demonstrated [49] that poly(*m*-phenylene vinylene-*co*-2,5-dioctyloxy-*p*-phenylene vinylene) (PmPV, see Table 1) effectively adsorbs onto the nanotube surface allowing to obtain a good dispersion of MWCNTs produced by arc discharge in toluene. Actually the method was used to purify the starting soot material from graphitic particles. Analogously, poly(*p*-phenylene vinylene), bearing cyano substituents, has been used to immobilize functional groups such as Zn-phthalocyanine onto the surface of SWCNT to generate active hybrid materials able to give, by photoexcitation, a metastable radical ion pair state, namely oxidized ZnPc and reduced SWCNT [50].

Vaisman et al. [51] have demonstrated that the dispersion of CNTs in water-insoluble polymers is favored by the presence of polymeric non-ionic surfactants containing a branched tail promoting a steric barrier that prevents aggregation and by unsaturated C–C bonds “securing” through π–π interactions the polymers to the Csp^2^ backbone of the nanotubes. Diblock (A–B) and triblock (A–B–A) copolymers demonstrated to be very efficient dispersing agents for CNTs thanks to the adsorption of one of the blocks onto the nanotube surface and the formation, by the other blocks dangling into the solution, of a steric barrier that prevents the CNT–CNT approach. Nevertheless, the prevailing effect among the two is still under investigation. Polyethylene glycol (PEG) chains with PEG repeating units as long as 8, have been demonstrated [52] to favor both self-assembly and dispersion of SWCNTs in chloroform and 1,2-dichlorobenzene once chosen a hydrophobic domain B (i.e., hexa-*p*-phenylene) able to establish π–π interactions with the B block of another monomer or with the SWCNT surface, respectively. On the other hand, the capability of polystyrene-*b*-polyisoprene (PS-*b*-PI) diblock copolymers to disperse MWCNTs seems to be dominated by the solvent selectivity of the block copolymers, being the direct interaction between the nanotubes and the polymers of secondary importance [53]. Indeed PS-*b*-PI demonstrated to disperse MWCNTs both in dimethylformamide (DMF), a polar solvent selective for polystyrene, and heptane, a non-polar solvent that is selective for the polyisoprene block.

Conjugated polymers like polyfluorene derivatives have been recently used for dispersing SWCNTs into different organic solvents. Poly[(9,9-dioctylfluorenyl-2,7-diyl)-*alt-co*-(6,6’-{2,2’-bipyridine})] (PFO-BPy, see Table 1) has been demonstrated [54] to selectively wrap highly semiconducting SWCNT species with large diameters ranging from 1.3 to 1.7 nm and to disperse both arc discharge and HiPco SWCNT in toluene and THF. The obtained dispersions were used to align and deposit semiconducting SWCNTs (s-SWCNTs) with exceptional electronic-type purity and at high deposition velocity (i.e., 5 mm min^−1^) with excellent control over the placement of stripes and quantity of deposited SWCNTs [55]. The rigid *non-wrapping* poly(arylenethynylene)s (PPEs) polymers (see Table 1), with backbone lengths less than 15 nm, have been demonstrated to make SWCNTs highly soluble in chloroform [56]. It is interesting to evidence that, depending of the bulkiness and/or the presence of ionic functional groups at the end of the side chains of PPE, it is possible to favor CNTs individualization or increase the adhesion between nanotubes via better π–π interactions eventually forming 2D “bucky papers”, respectively [56].

Nguenda et al. demonstrated [57] for the cholesterol moiety of a multivalent cholesterol-containing polymer (CEM-EHA, see Table 1) an interaction very similar to that between pyrene and carbon nanotubes with cholesterol laying flat above the graphene surface. Supramolecular complexes of these copolymers with CNTs were soluble in THF, toluene and isooctane.

Supramolecular complexes can be formed between CNTs and a biopolymer such as DNA, in order to disperse CNTs in aqueous solutions (see next section). Recently two decanoyl-functionalized guanosines (LipoGs, see Table 1) have been used [58] for the disaggregation of SWCNTs in chloroform. Different spectroscopic and microscopic methods highlighted a well debundling of SWCNTs whereas fluorescence measurements showed the strong adsorption of LipoGs onto the CNT surface. The authors demonstrated that the ability of the investigated LipoGs to disperse SWCNTs depends on their tendency to self-assemble into ribbon-like supramolecular structures that wrap, although not specifically, around the nanotube. It is interesting to note that the self-aggregation properties of the dispersant are crucial as the tendency of super-aggregate into bigger fibers can favor their desorption from CNT surface and thus CNTs bundling.

### Non-covalent interactions for solubilizing CNTs in water

In order to favor the dispersion of CNTs in water the widely and most used approach is the adsorption of surfactants onto the nanotube sidewalls. Their main advantages are their cheapness, their ready commercial disposability and simplicity of use. These small molecules have typically a hydrophobic tail and a hydrophilic head group, the former is intended to favor adsorption onto the hydrophobic nanotube walls and the latter to promote affinity with the aqueous bulk solvent. Indeed, surface active agents can modify the features of the interface between CNTs and the bulk medium providing additional repulsive forces, either electrostatic or steric, and decreasing the surface energy. In 1997 Bonard et al. [59] purified CNTs from graphitic nanoparticles by sonicating CNTs for a few minutes in a 1% aqueous solution of an ionic surfactant such as sodium dodecyl sulfate (SDS). The chemical adsorption of SDS molecules on the surface of the nanotube induces electrostatic repulsion between polar heads that expose in the aqueous solution thus preventing CNTs aggregation and inducing the formation of stable aqueous black suspensions.

**Figure 2:**
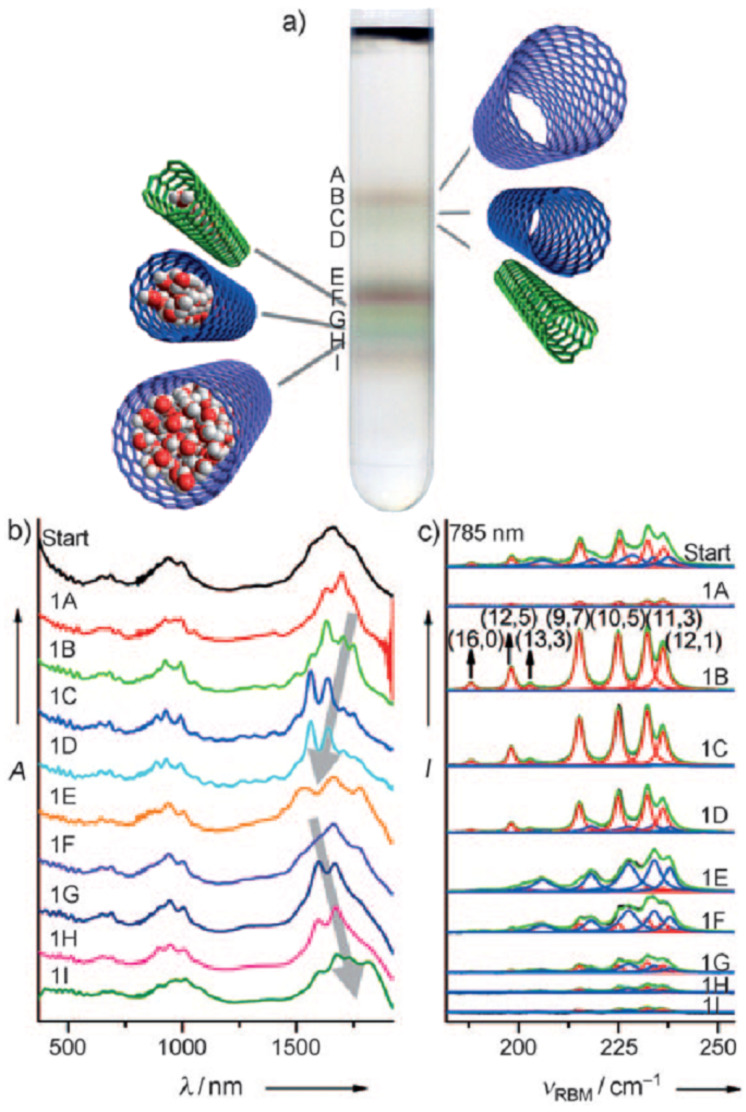
Sorting of empty and water-filled Arc SWCNTs (2% w/v SC). a) Centrifuge tube containing sorted Arc SWCNTs; b) Absorption spectra of the original solution and of the sorted fractions; c) Resonance Raman spectra of the different fractions excited at 785 nm. Reprinted with permission from [62]. Copyright 2011, Wiley VCH.

During the years stable aqueous CNTs dispersions were obtained with differently charged and non-ionic commercial surfactants such as sodium dodecylbenzen sulfonate (SDBS), cetyltrimethylammonium *p*-toluenesulfonate (CTAT), cetyltrimethylammonium bromide (CTAB) and sodium cholate (SC) enhanced by sonication [60] (see Table 2). The most efficient dispersant, SDBS, was able to disperse as much as 0.06% w/w SWCNTs in water [60]. Very recently Oh et al. [61] determined that binding strengths of surfactants to SWCNTs follows the trend SDBS > SC ≈ Flavin mononucleotide (FMN, see Table 2) > SDS, irrespective of electronic types of SWCNTs. SC were demonstrated to be particularly efficient in dispersing CNTs also in the absence of any sonication. For this reason SC has been used in cases where integrity of carbon nanotubes has to be ensured, i.e., to sort water-filled and empty CNTs via ultracentrifugation to equilibrium by exploiting a density gradient (see Figure 2) [62].

**Table 2:** Some amphiphilic molecules used for the dispersion of CNTs in water.

dispersant (structure or name)	acronym	ref.

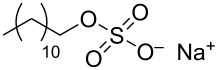	SDS	[59–61,63–65,68]
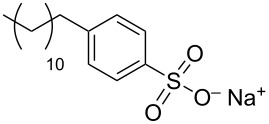	SDBS	[60–61,68]
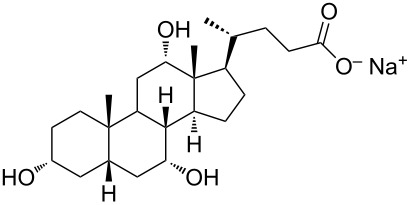	SC	[60–62]
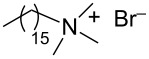	CTAB	[60]
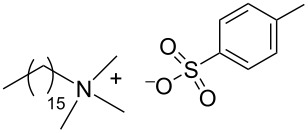	CTAT	[60]
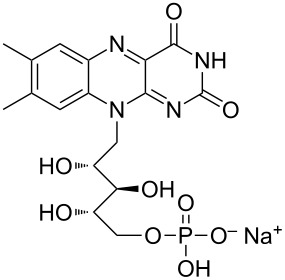	FMN	[61]
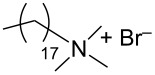	ODTABr	[63]
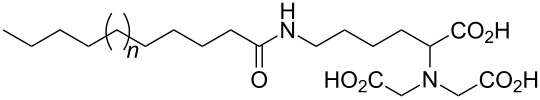	NTA	[63]
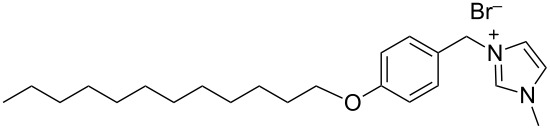	T3	[67]
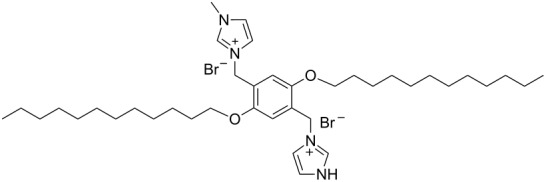	T1	[67]
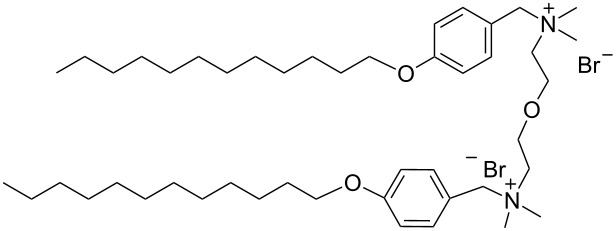	T4	[67]
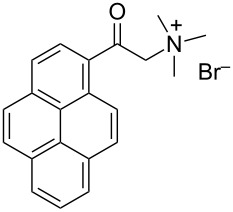	–	[72,76]
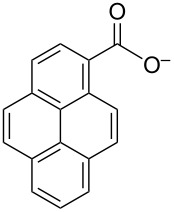	–	[73]
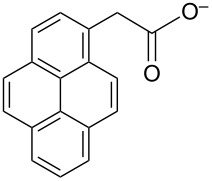	–	[73]
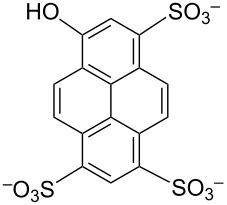	–	[73]
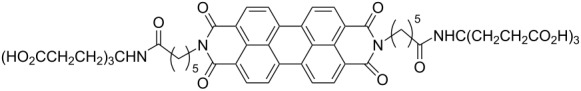	perylene dye	[74]

**Figure 3:**
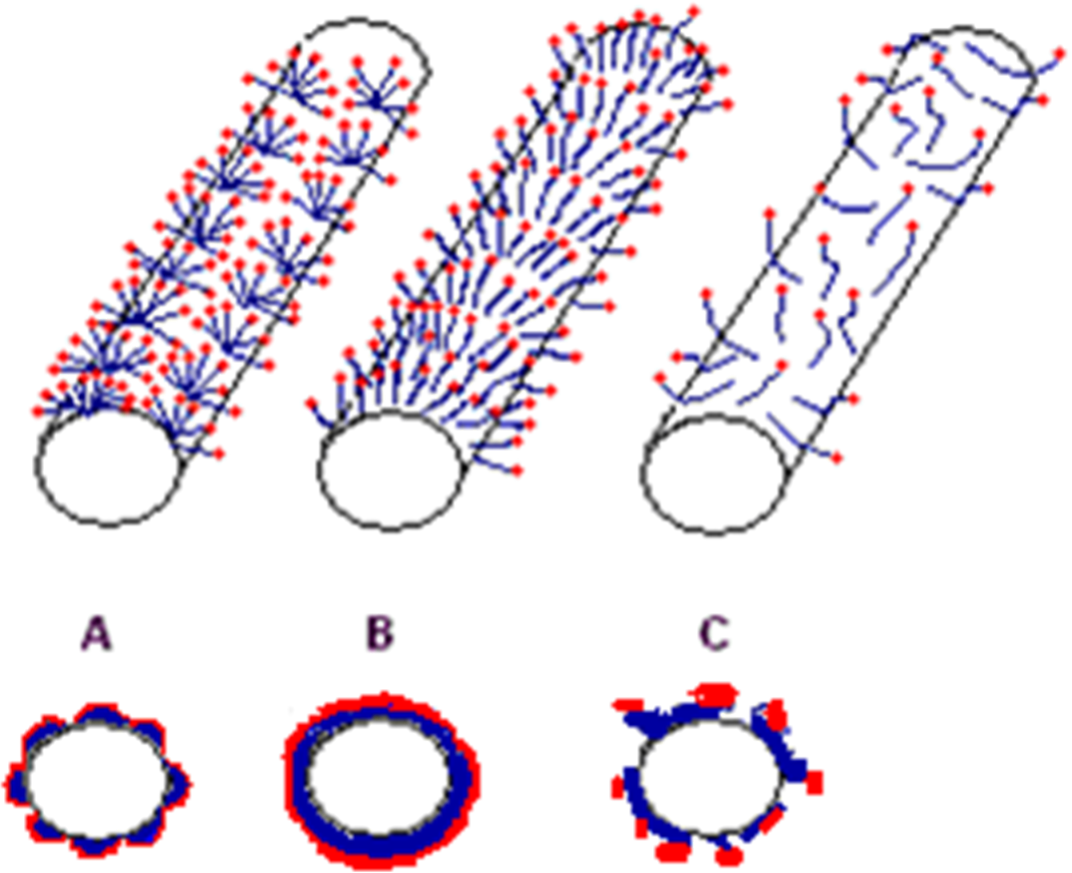
Schematic illustration of the different possible organizations of surfactant molecules on the surface of a CNT. Upper images refer to lateral projections; lower images refer to cross sections.

Different papers have faced the possible organizations of surfactant molecules on the surface of CNTs and still several conflicting evidences are reported in the literature. Richard et al. were among the first authors envisaging the assembling of SDS on the nanotube surface [63]. They discovered, through TEM analysis, that surfactants characterized by long alkyl chains, either SDS, cationic octadecyltrimethylammonium bromide (OTABr) and nitrilotriacetic acid substituted with a single lipidic saturated chain of 10, 12, 14, or 18 carbon atoms (NTA) do arrange into half-cylinders oriented parallel to the tube axis (see Figure 3, arrangement A) highlighting the crucial role of supramolecular assembling for CNT-dispersant interactions.

The environment of nanotubes in SDS micelles was modeled also by O’Connell et al. [64] through molecular dynamics simulations run for 0.4 ns. The nanotube represented the core of a SDS cylindrical micelles were the hydrophobic tails of SDS molecules could adopt a wide range of orientations with respect to the tube (see Figure 3, arrangement B). As no water density was observed in the proximity of the tube, they speculated that the individual nanotubes were essentially in a pure hydrocarbon environment. On the other hand, SANS data, performed by Yurekli et al. [65] strongly suggested that the dispersion of SWCNTs by SDS is due to the formation of a structureless, adsorbed layer of surfactant on the individual SWCNTs (see Figure 3, arrangement C).

Analogously, we have evidenced [66–67] that gemini surfactants, characterized by two alkyl chains and two polar head groups separated by a spacer in a single molecule, are relatively effective dispersants also below their CMC or at very low surfactant concentrations confirming their compact alignment on the nanotube surface. Indeed, a gemini surfactant ensures (i) doubled hydrophobic interactions with the CNT sidewalls, due to the presence of twin chains rather than a single alkyl tail and (ii) favorable packing, thanks to the spacer that forces the twin ionic groups to reside in a space-filling geometry reduced with respect to that of two distinct single-chained surfactant molecules. Similarly, Zhong and Claverie demonstrated from adsorption isotherm measurements [68] that MWCNTs form stable aqueous dispersions when 90% of their surface is covered by the dispersant, while for SWCNTs, 45% of the surface has to be covered in order for the dispersion to be stable. Nevertheless, they also showed that a good dispersion is the result of a delicate compromise, i.e., at high surfactant concentrations a significant amount of the surfactant, at least more than 40%, is free in solution and can reduce the Debye length thus destabilizing the colloidal suspension. Analogously, Bonard et al. [59] evidenced that CNTs bundles form in the presence of a large excess of SDS surfactant (i.e., well above the CMC) due to the depletion interaction induced by the surfactant micelles on large colloidal particles.

**Figure 4:**
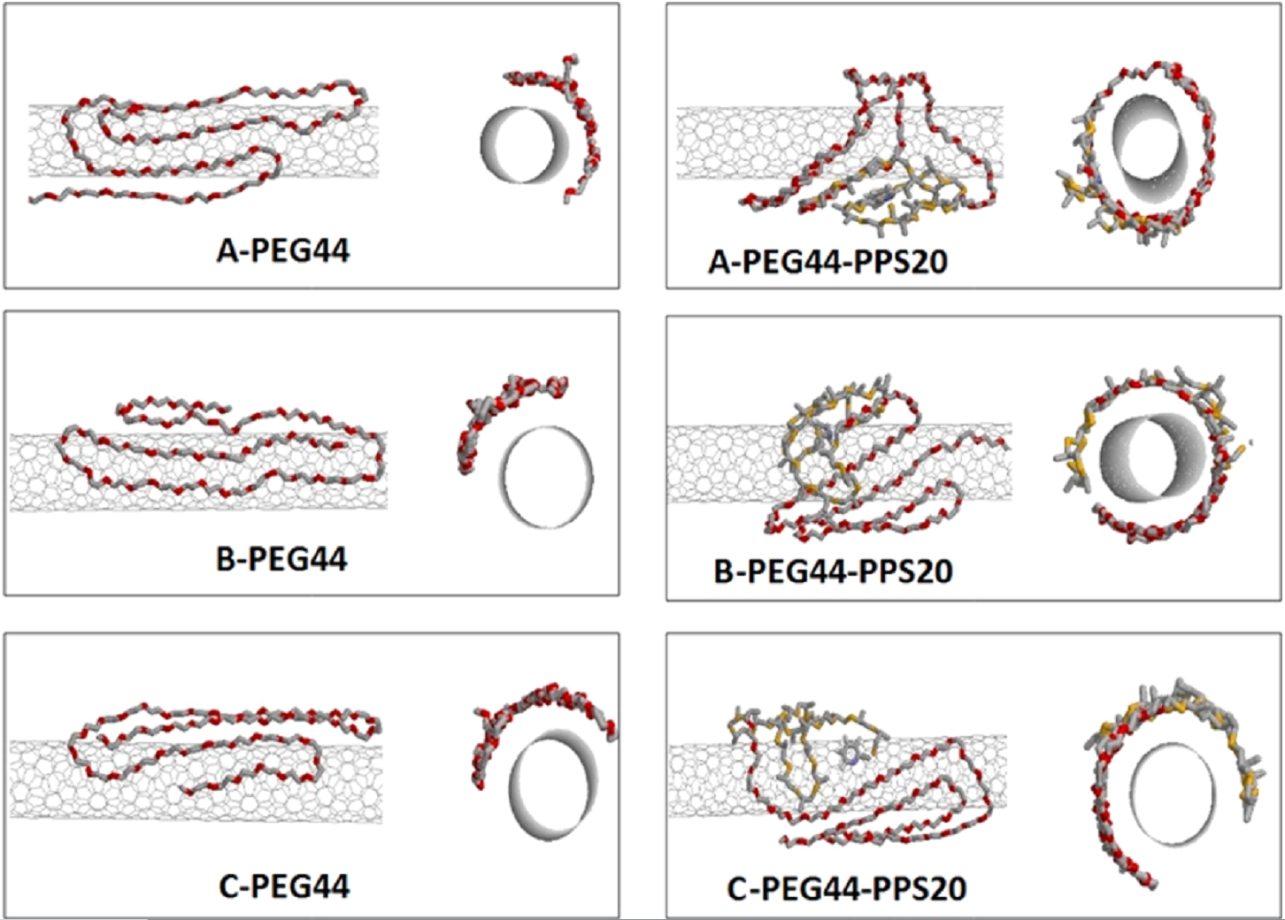
Representative structures of poly(ethylene glycol), PEG_44_, and poly(ethylene glycol-*bl*-propylene sulfide), PEG_44_−PPS_20_, adsorbed on SWCNT as obtained by essential dynamics analysis. The presence of PPS (see structures on the right) enables the block copolymer chain to fully wrap the CNT thus minimizing nanotube−nanotube aggregating interactions. Reprinted with permission from [71]. Copyright 2012, American Chemical Society.

Examples of polymers that favor CNTs dispersion in water are countless. This review is not intended to list all of the investigated polymers, nevertheless it is worth mentioning that the majority of polymers and block copolymers have been demonstrated [69] to wrap the nanotube exposing their polar domains towards the aqueous environments while favoring the contact of their hydrophobic domains with the nanotube surface. The wrapping of the SWCNTs by water-soluble polymers is a thermodynamically driven phenomenon because the free energy cost of forcing the polymer into a regular wrapping around the nanotube appears to be well counterbalanced by the reduction of the hydrophobic penalty consequent to the contact of CNT hydrophobic surface with the surrounding aqueous medium. Also in the case of polymers and similarly to what has already been underlined for classical surfactant molecules, we have demonstrated [70] that polymeric micelles concentration is not related to the formation of stable SWCNT dispersions, the outmost requirement in order to obtain a stable CNTs dispersion being the adsorption degree, up to an almost complete saturation, of the nanotube surface with proper dispersant molecules. Molecular dynamic simulations allowed us to highlight that the SWCNT surface coverage is systematically ensured by the hydrophobic domain of the amphiphilic dispersant. An increase of hydrophobicity therefore causes weaker inter-tube contacts, makes CNTs bundles precipitation more difficult, dehydrate the CNT interface and reduce water ordering around the CNTs [71] (see Figure 4). Unlikely, in none of our simulations we have observed hydrophilic chains extending and swelling in the aqueous environment. Nevertheless, our simulations considered always available SWCNT surfaces higher than the potential coverage area of the polymer [71].

Noteworthy are the numerous examples of PAH functionalized with hydrophilic domains used both to solubilize CNTs in water and to non-covalently functionalize CNTs with proper functionalities [72–73]. A perylene dye (see Table 2) has been used to exfoliate and suspend SWCNTs through strong π–π interactions in water. This hybrid system granted charge transfer chemistry of pristine SWCNTs used as an unusual electron donor towards the electron acceptor molecule perylene [74].

Regarding biocompatible polymers, the most representative one is DNA and different examples of non-covalently functionalized SWCNTs with DNA have been published. While double-stranded DNA (dsDNA), being more rigid, displayed a less efficient wrapping, single-stranded DNA (ssDNA) has been used to disperse CNTs or to separate and purify them. Indeed, by sonicating ssDNA with HiPco SWCNTs for ca. 1 h a solution as concentrated as 25 mg/L has been obtained [75]. These functionalized SWCNTs have proved to be able to internalize oligonucleotide chains into living cells whereas ssDNAs have been translocated into cell nucleus upon endosomal rupture triggered by NIR laser pulses. Continuous NIR radiation can cause cell death because of excessive in vitro local SWCNTs heating [75]. In these carbon scaffolds the nucleobases directly interact with SWCNT sidewalls, while the hydrophilic sugar-phosphate backbone is oriented towards the solvent [5].

### Design of novel dispersant

At this point we have all the hints for properly designing an efficient dispersant for both aqueous and organic solvents. Different authors have already tried to improve the dispersing ability of commercial organic molecules in order to prepare stable CNTs dispersions. Tomonari et al. [76–77] described the design of ammonium-substituted polycyclic aromatic compounds (see Table 3) and showed the better performance of polycyclic aromatic moieties with respect to simple phenyl or naphthyl groups in favoring SWCNTs dispersions.

**Table 3:** Novel designed dispersants.

dispersant	acronym	ref.

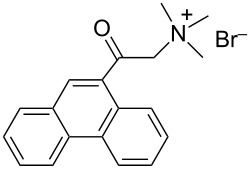	–	[76]
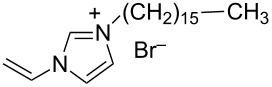	hvimBr	[79–80]
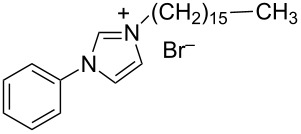	hphimBr	[80]
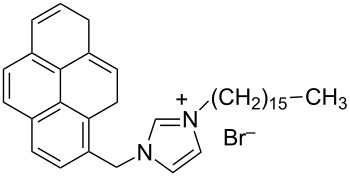	HpymimBr	[80]
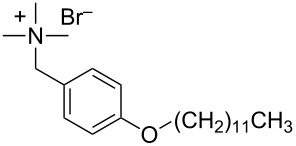	pDOTABr	[66]
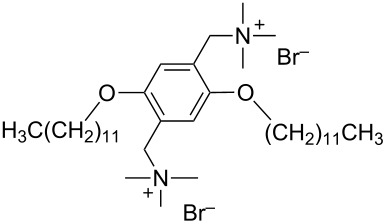	pXDo(TA)_2_Br	[66]
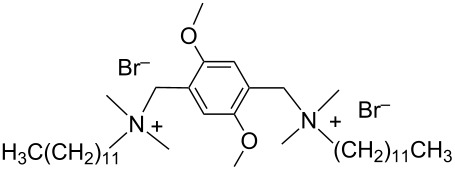	pXMo(DDA)_2_Br	[66]

In the last five years we have synthesized different surfactants with the aim to increase the affinity of the dispersant for the nanotube surface or favor its hydrophilicity. Indeed, we have demonstrated that, in order to increase the efficiency of dispersants, is essential to reach an optimal hydrophobic/hydrophilic balance which favors adsorption onto the nanotube sidewalls over self-assembly. Exploiting the excellent capacity of imidazolium-based ionic liquids [78] to disperse CNTs, we conceived an ionic liquid-based surfactant, 1-hexadecyl-3-vinylimidazolium bromide (hvimBr, see Table 3) which demonstrated to be more effective than SDBS at high surfactant concentrations [79]. Despite the introduction in the polar head of the ionic liquid-derived surfactant of aromatic groups (i.e., benzene in hphimBr and pyrene in hpymimBr, see Table 3) enhances the affinity for SWCNTs, as confirmed by molecular dynamic simulations, it reduces the solubility of the surfactants in water and renders them more prone to self-assemble [80]. Starting from a good SWCNT dispersant such as *N*-[*p*-(*n*-dodecyloxybenzyl)]-*N*,*N*,*N*-trimethylammonium bromide (pDOTABr, see Table 3) we improved [66] its dispersing capability by preparing the corresponding gemini counterparts [66]. Indeed, the novel surfactants, namely 2,5-bis(*n*-dodecyloxy)-1,4-bis(*N*,*N*,*N*-trimethylammoniomethyl)phenyl bromide [pXDo(TA)_2_Br] and 2,5-dimethoxy-1,4-bis[*N*-(*n*-dodecyl)-*N*,*N*-dimethylammoniomethyl]phenyl bromide [pXMo(DDA)_2_Br] (see Table 3), demonstrated to disperse SWCNTs at much lower surfactant/CNTs weight ratios with respect to conventional surfactants (i.e., 3 times lower than CTABr, 2 times lower than SDBS and 6 times lower than SDS). Besides their capacity to enrich the dispersed mixture in semiconducting tubes paves the way for applications in CNT fractionation. Analogously, the substitution of the ammonium groups with imidazolium moieties in pDOTABr ensured an increase of the water solubility of the corresponding surfactant and a consequent improvement of their CNTs dispersing ability [67].

### Reversibility: the advantage of non-covalent functionalization

The non-covalent functionalization of carbon nanotubes, aside from preserving CNTs conjugated double bonds, has another feature that makes this approach even more interesting, which is the reversibility. Indeed, it is possible to regain the bare CNTs from the dispersant-coated counterparts both in aqueous or organic solvents. Among the numerous papers reporting on the subsequent functionalization and functionalization removal of CNTs we review here the main examples in which such an approach is useful for purification, enrichment or separation of different types of nanotubes and extensive potential applications in different fields. An intelligent way to exploit at the same time the strong and selective interactions of many CNT dispersants and their ability to reversibly desorb from the CNT surface is the use of molecules capable of responding to different specific external stimuli [81] such as variation of the solvent, pH, temperature, light and redox conditions. Interestingly, many biomolecules have this capability, providing a viable alternative when reversibility of these processes is preferred.

**Solvent variation.** The first and easiest method to remove the non-covalent functionalization is to change the solvent system [82]. Anyway, due to the strong interactions with the nanotube sidewalls, the removal of the dispersant via solvent washing is often a difficult task as in the case of some polymers [83]. Lellouche et al. proposed to exploit the non-covalent interaction of neutral iron tricarbonyl complexes with MWCNTs to detect their sidewall oxygenated defects. A simple CH_3_CN washing of the composite allows the complete desorption of the adsorbed complexes [84].

A similar result was obtained by adding CH_3_CN to CNTs dispersed in chlorinated solvents by using foldamers (i.e., synthetic and designable oligomers) [85]. In this case, CH_3_CN did not act as a simple “washing” solvent but rather induced a variation of the conformation of the foldamers due to the solvophobic effect associated to the increase of solvent polarity on passing from chlorinated solvents to CH_3_CN.

A recent and interesting way to get a reversible functionalization of MWCNTs makes use of molecules that self-assemble around the nanotube forming supramolecular polymers. Llanes-Pallas et al. have shown that such a non-covalent solubilization can be realized by exploiting the formation of hydrogen bonds between the 2,6-di(acetylamino)pyridine and the imidic groups of uracil derivatives [86]. In apolar organic solvents such hydrogen bonding interactions are effective and keep the nanotube in solution, whereas the addition of polar solvents destroys the H-bonds causing the detachment of the polymer from CNT surface and the precipitation of the nanotubes.

**pH responsive systems.** It is possible to control the polymer-nanotube affinity, and consequently the CNTs aggregation/disaggregation state, by varying the proton concentration. An effective method of CNTs purification takes advantage of the use of 1-pyreneacetic acid (Py-COOH). This acid, with a high affinity to the nanotube surface thanks to the presence of pyrene, deprotonates under basic conditions and becomes able to solubilize and separate the nanotubes from carbonaceous impurities in water. The acidification of the solution to the original acid causes the precipitation of the nanotubes. Nevertheless, in order to recover the bare nanotubes they must be thoroughly washed with deionized water and ethyl acetate in repeated dispersion–centrifugation cycles, and eventually refluxed in ethyl acetate for 12 h [87].

Some polymers are able to undergo conformational changes in response to a pH change, basically due to a protonation or deprotonation step. Thus, Liang et al. [88–89] have recently studied a conjugated tetrathiafulvalene vinylogue-fluorene copolymer able to selectively disperse low-diameter semiconducting SWCNTs in organic solvents. Upon addition of trifluoroacetic acid to the dispersion a rapid precipitation of completely naked and extremely pure SWCNTs can be observed due to desorption of the polymer from the nanotube walls.

Single-stranded DNA is able to disperse SWCNTs in water at pH 7 and shows a selective affinity towards semiconducting CNTs. Arnold et al. demonstrated [90] an easily scalable and automatable method for the enrichment of SWCNTs by diameter size using density gradient ultracentrifugation. Isolated DNA-wrapped semiconducting SWCNTs are separated into colored bands according to the buoyant density. Besides, as the pH of the aqueous environment controls the secondary structure of DNA, a precipitation/redispersion sequence can be obtained upon simply lowering the pH below 4–5 and raising it to 7. The process is totally reversible, nondestructive and can be repeated several times [91].

Worth noting is also the pH response of poly-L-lysine (PLL)/SWCNTs complexes to pH variations. At pH values higher than 9 the aqueous dispersions of PLL/SWCNTs complexes lose their stability due to the different affinity of PLL towards the nanotubes in their neutral or charged state. Indeed, in aqueous solutions at pH < 9 PLL interacts with the nanotubes either via hydrophobic interactions between the PLL hydrocarbon linker moieties (–C_4_H_8_–) and the SWCNT surface and/or cation–π interaction between protonated amine groups and the nanotube π-electron system. Both interactions are affected by pH variation as PLL assumes an α-helix conformation at high pH but adopts an uncoiled conformation in acidic or neutral pH, whereas ammonium groups deprotonate at pH higher than 9 [92].

Another example is the polymer poly(acrylic acid) used as CNT dispersant. Upon changing the pH of the aqueous solution its charges, its ability to form hydrogen bonds and conformations may vary thus conferring to the polymer a different dispersing power [93].

Another example of reversible dispersion/precipitation of CNTs is the folic acid (FA)/SWCNT system. The insolubility of folic acid in acidic aqueous solutions prevents it acting as a CNT dispersant. Nevertheless, once CNTs have been FA-coated, the simple basification of the mixture results in a complete CNT redissolution without the need for sonication [94].

Guo et al. have shown that using CO_2_-responsive polymers is possible to tune the dispersion and aggregation of SWCNT in organic and aqueous media [95]. The amidine groups of the adsorbed polymers in the presence of CO_2_ do protonate thus leading to an increased polymer–polymer electrostatic repulsion and to the disassembly of the bundles. CNTs precipitation is induced by N_2_ bubbling and CO_2_ displacement.

**Temperature responsive systems.** The precipitation of a FA-coated SWCNT system can be controlled also through temperature variation. The simple heating of the dispersion at 80 °C for 20 minutes causes the peeling of the FA from the SWCNT surface and induces their precipitation. The drawback of this precipitation strategy is that, in order to regenerate the dispersion, it is mandatory to sonicate the sample [94].

Wang et al. showed that, upon increasing the temperature, poly(*N*-isopropylacrylamide) passes from the enthalpically favored to the entropically favored conformation leading to the precipitation of the initially dispersed SWCNTs [92]. The process is reversible and a 2 minutes sonication at 0 °C is enough to regenerate the dispersion. Etika et al. employed a thermo-responsive poly(*N*-cyclopropylacrylamide) copolymer with 5 mol % pyrene side groups to stabilize aqueous SWNT suspensions. At temperatures above the lower critical solution temperature of the copolymer, the conformation of the dispersant changes reducing the steric layer thickness on the CNT surface and inducing precipitation [96].

**Light responsive systems.** Another very interesting phenomenon is the light-controlled dispersion and reaggregation of SWCNTs in solution. This means realizing a change of the state of aggregation of nanotubes controlled by simple irradiating the sample under UV–visible light.

Chen et al. pointed out that the UV photoirradiation of well-dispersed SWCNTs coated with a poly(ethylene glycol)-terminated malachite green derivative is responsible for the CNTs reaggregation [97]. Indeed, UV light irradiation converts the dispersant into a cation with a superhydrophilic nature and thus more prone to interact with water than with the nanotube surface. The CNTs dispersing ability of azobenzene polymers is also tunable by the light-promoted *trans–cis* isomerization of azobenzene units that allow to switch the polymer conformation from a wrapping to an unwrapping state [98]. Shiraki et al. have shown that NIR laser irradiation coagulates a modified helical polysaccharide/SWCNT complex [99]. Recently, a UV-responsive supra-amphiphile based on the host–guest complexation of a water-soluble pillar[6]arene and a 2-nitrobenzyl ester derivative has been used to control the dispersion behavior of MWCNTs. After 30 minutes UV light irradiation CNT aggregate due to photocleavage of an hydrophilic segment from the hybrid [100].

**Redox responsive systems.** Oxidation or reduction of the dispersant can as well be favorably exploited for CNTs precipitation/dispersion cycles. Ortiz-Acevedo et al. obtained a diameter-selective solubilization of SWCNTs by using cyclic peptides containing thiol groups [101]. The oxidation induces polymerization of the peptides which wrap around the nanotube achieving a size-selective dispersion. The precipitation of SWCNTs is induced by reduction of newly formed disulfide bonds. Other experimental evidences have been reported on reversible nanotube dispersion via modulation of the oxidation state of a metal complex used as the dispersant. Nobusawa et al. demonstrated how the addition of ascorbic acid to a chloroform solution of SWCNT coated with a Cu(II) complex, in which the 2,2’-bipyridine derivative ligand bears two cholesteryl groups, induces the precipitation of the nanotubes due to the formation of the reduced Cu(I) complex [102]. The conformational change from the planar structure of the Cu(II) complex to the tetrahedral structure of the Cu(I) complex prevents π-stacking interactions between the complexes and the SWCNT surface. Moreover, the precipitate can be redissolved in solution by bubbling air into the mixture. Some foldamers, able to wrap and solubilize nanotubes, unfold and dissociate from the nanotube surface leading to CNTs precipitation upon oxidation [103].

**Adsorption–desorption induction.** Non-covalent functionalization of CNTs can be controlled by an adsorption–desorption equilibrium. As already highlighted above, when polymers are used as the dispersants some free polymer is always present in the solution. It has been demonstrated that too much free polymer in the bulk solution can lead to aggregation of the CNTs because of the depletion effect. As an example, Meuer et al. showed [104] that CNTs dispersed by pyrene-substituted poly(methyl methacrylate) do precipitate on addition of further polymer.

The adsorption–desorption equilibrium is a real critical point when dealing with dilution steps (i.e., administration of drug targeting SWCNTs in the blood stream) as it is important to ascertain that a proper amount of the dispersant keeps covering the nanotube surface even under very diluted states [105].

Non-covalent functionalization can be reversed also by means of thin-layer chromatography (TLC) experiments by exploiting the different affinity of the dispersant for the stationary or the mobile phase [106].

## Conclusion

The reported findings highlight how it is possible to obtain stable and relatively concentrated dispersions of CNTs both in organic and in aqueous solvents. Once CNTs are properly disentangled by overcoming the strong van der Waals interactions among the tubes via sonication, it is essential to disfavor the approach of CNTs to each other. Thus, it is possible to simply obtain organic CNTs dispersions by exploiting synergistic effective polar and π-stacking interactions of CNTs with elected solvents such as NMP. A second and widely used approach involves the addition of proper molecules characterized by the capability to interact both with the nanotube surface (i.e., mainly through π-stacking and van der Waals interactions) and with the surrounding medium. Among the most used dispersants, we may recall PAH, porphyrines and lipophilic polymers for dispersion in organic solvents whereas surfactants, DNA and block copolymers are mainly used for dispersion in aqueous solutions. The possibility of processing CNTs with the use of proper dispersing agents and/or to control their bundling through external stimuli such as pH, temperature, redox reactions or light irradiation, thanks to the reversibility of non-covalent functionalization, represents nowadays one of the major challenges in overcoming their limited manipulability and therefore in developing applications for the future.
